# Rectangular loop suture to correct iris capture of the posterior chamber intraocular lens

**DOI:** 10.1186/s12886-020-01650-3

**Published:** 2020-09-25

**Authors:** Ke Lin, Zhixiang Hu, Zhong Lin, Tianyu Chen, Yongping Tang, Ronghan Wu

**Affiliations:** Eye Hospital and School of Ophthalmology and Optometry, Wenzhou Medical University, 270 Xueyuan Road, Wenzhou, 325000 Zhejiang Province China

**Keywords:** Iris capture, Posterior chamber intraocular lens, Rectangular loop suture

## Abstract

**Background:**

To report a new technique for iris capture of the posterior chamber intraocular lens (IOL) implanted in patients with a posterior capsule defect.

**Methods:**

In this retrospective case series, a rectangular loop ciliary body suture technique was performed to rectify iris capture. The suture passes between the IOL and iris in a direction perpendicular to the iris edge capturing the IOL.

**Results:**

A total of three IOLs with iris capture underwent a rectangular loop suture technique. No recapture was observed postoperatively. In one case, large astigmatism appeared after the surgery but recovered at 1 month post operation. No further complications were found.

**Conclusions:**

The rectangular loop suture technique is an effective, convenient, and minimally invasive method for iris capture of the IOL.

## Background

In eyes with a capsular defect, intracapsular intraocular lens (IOL) implantation is not possible. Accordingly, several alternative surgical techniques can be applied, including transscleral IOL fixation, sutureless intrascleral fixation, and the scleral hook technique [1–3]. While these have proven to be safe and effective, some complications remain, including suture breakage, IOL tilt or dislocation, haptic slip, and iris capture [4–6]. Cho and Yu reported an incidence of iris capture of 23% in patients who underwent combined scleral IOL fixation and pars plana vitrectomy. Iris capture leads to blurred vision, photophobia, and dull pain. Moreover, iritis and secondary glaucoma may occur during long-term iris capture [7].

Iris capture of the IOL may be caused by pupil dilation [8], IOL deviation or tilt, a floppy iris [9], or reverse pupillary block [10]. Although pupil dilation or laser iridotomy of a reverse pupillary block are useful for iris capture, their success rates are relatively low [11–13]. When iris capture is not resolved by these techniques, surgical techniques can be applied, including IOL repositioning [12] and exchange [14]. However, these surgeries cause relatively large injuries and can result in bleeding and corneal endothelium loss. We performed a minimally invasive technique for three cases, and no iris recapture was observed post surgery.

## Methods

### Surgical technique

**Fig. 1:**
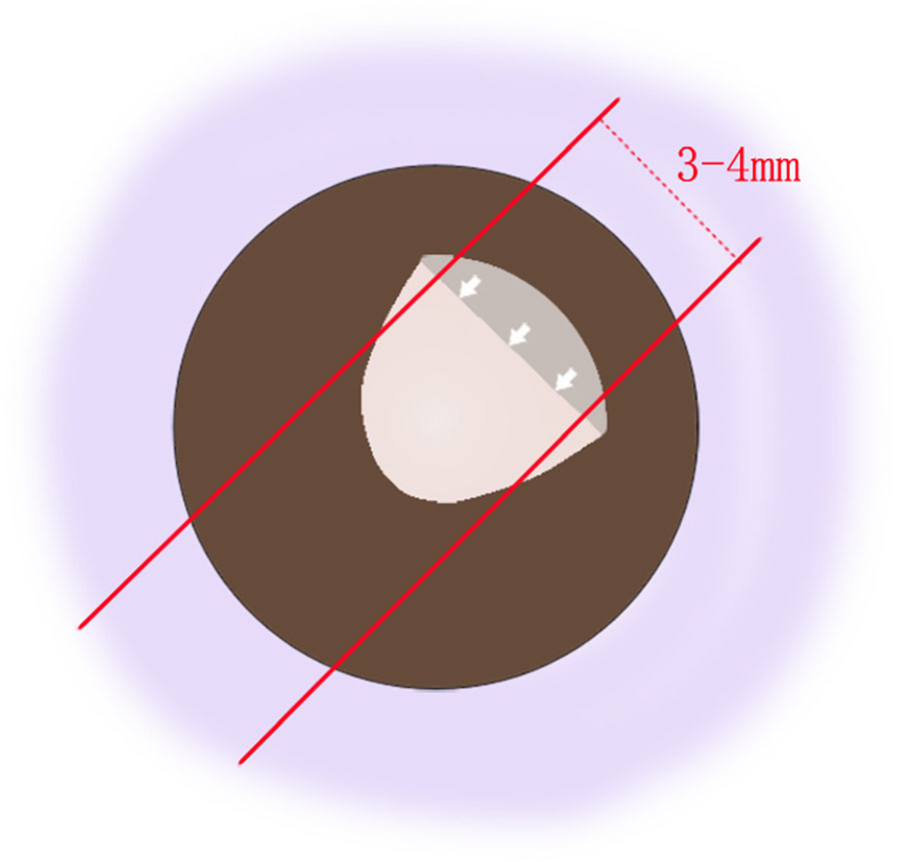
Design of suture direction. The white arrows indicate the iris edge capturing the IOL, the red lines show the direction of the sutures, with the distance between the two lines of 3–4 mm

**Fig. 2:**
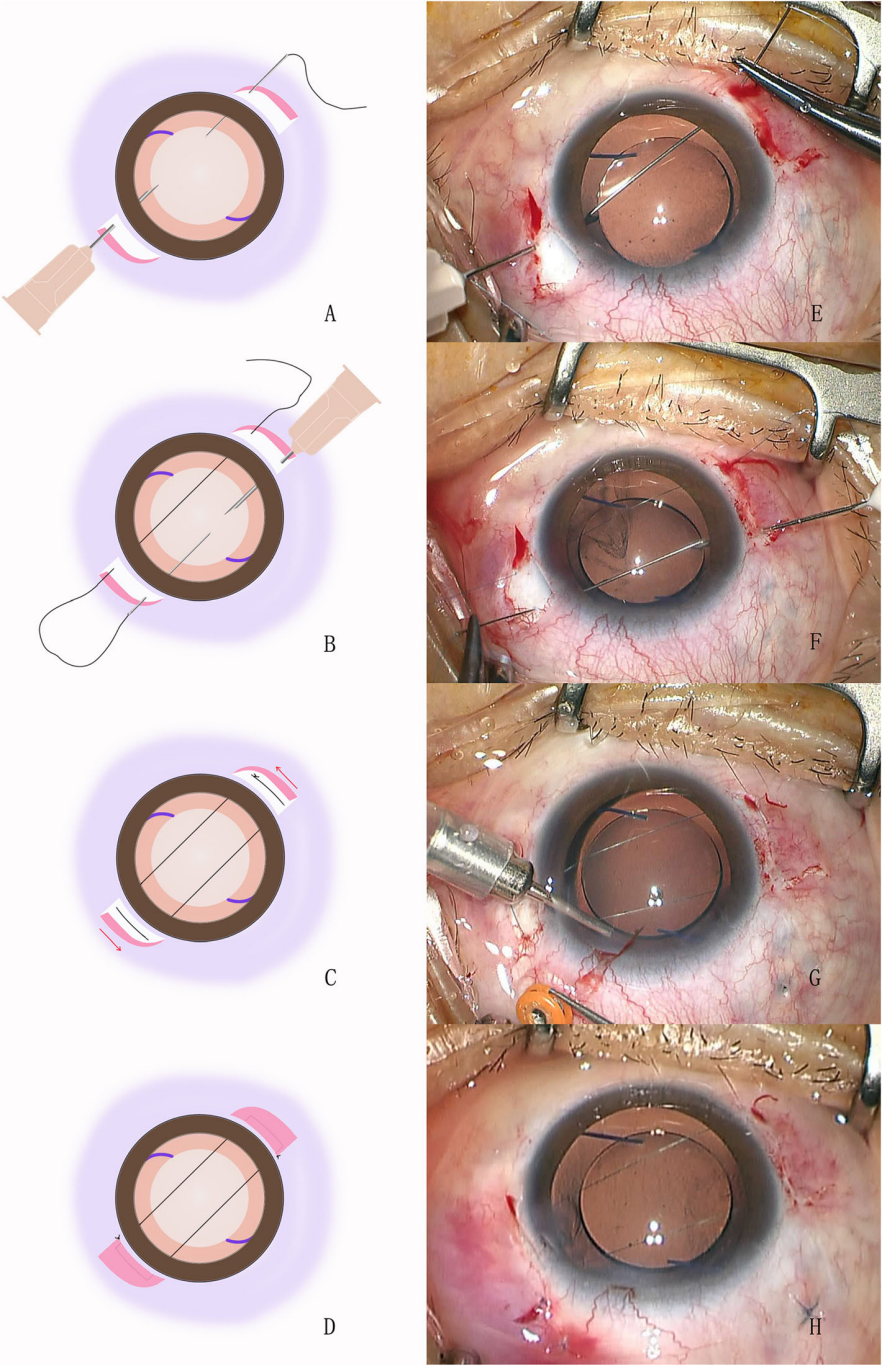
Surgical procedures. **a**: The needle is inserted into the posterior chamber between the iris plane and IOL optic, and is withdrawn with the assistance of a 26-gauge needle. **b**: The needle is inserted at 7 o’clock and withdrawn at 2 o’clock in the same manner. **c**: The suture is knotted and the knot is buried by rotating the loop. **d**: The conjunctival incisions are sutured. **e**-**h**: The figures captured from the intraoperative video recording are shown. Additional vitrectomy was performed for this patient to remove the residual vitreous and intraoperative hemorrhage

First, we design the route of sutures according to the appearance of patient’s anterior segment. The sutures are perpendicular to the iris edge capturing the IOL preoperatively. For example, the direction of sutures in Fig. 1 is 7:30 to 1:30 o’clock. After retrobulbar anesthesia, conjunctival incisions are made at the limbus from 1 to 2 o’clock and 7 to 8 o’clock to expose the sclera areas where the needles are going to be inserted through. Nonabsorbable 10–0 nylon suture with long straight needles is used. The needle is inserted into posterior chamber 2 mm behind the limbus at 1 o’clock, between the iris plane and IOL optic, and is externalized at 8 o’clock 2 mm behind the limbus with the assistance of a 26-gauge needle (Fig. 2a). Subsequently, the needle is inserted at 7 o’clock and withdrawn at 2 o’clock in the same manner (Fig. 2b). The suture is knotted and the knot is buried by rotating the loop into the vitreous cavity to avoid postoperative conjunctival irritation (Fig. 2c). The conjunctival incisions are sutured with 8–0 absorbable thread (Fig. 2d).

## Results

### Case 1-left eye

**Fig. 3:**
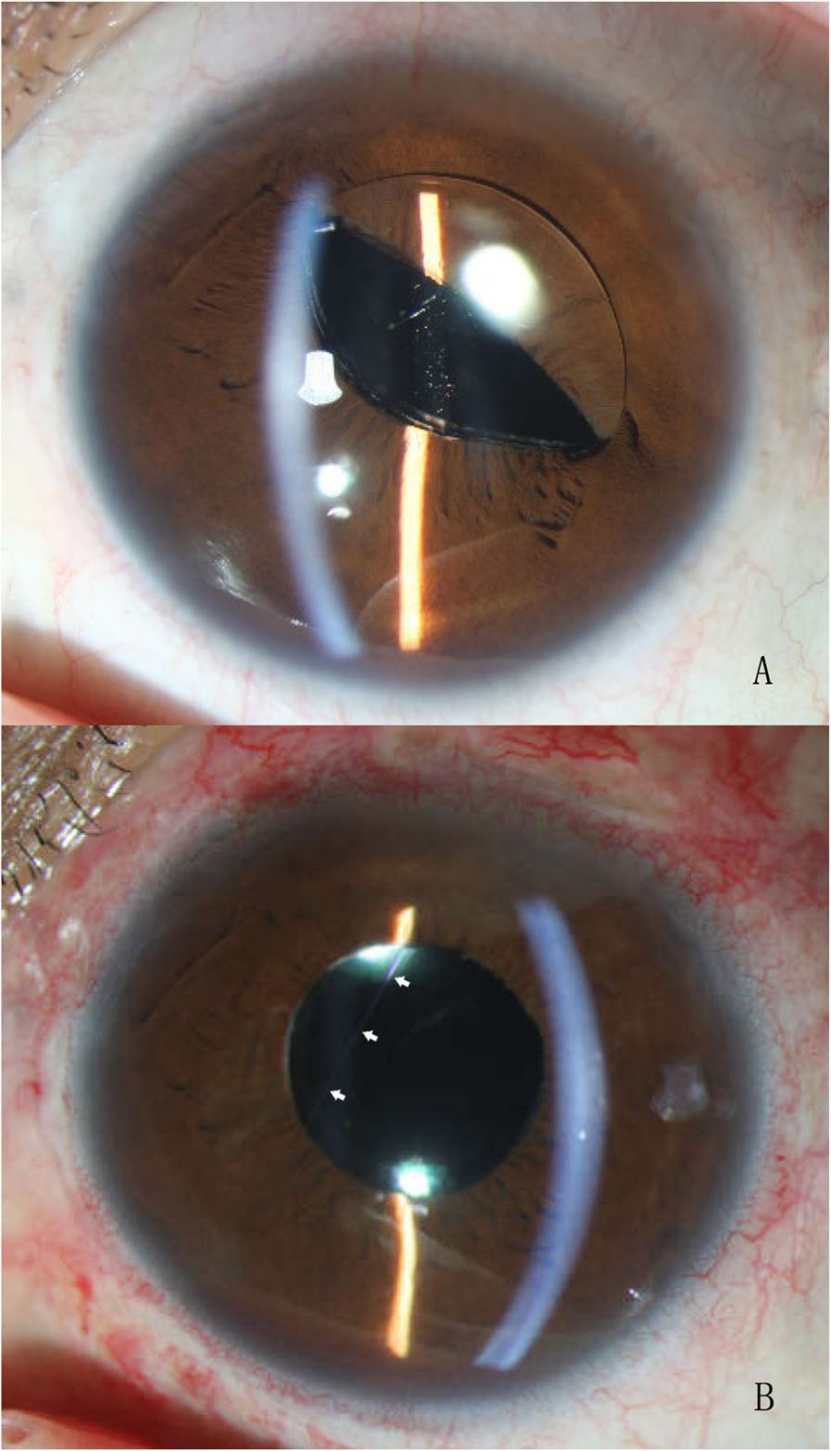
The left eye in case 1: pre- (**a**) and postoperative (**b**) anterior segment photography. The white arrows indicate part of the suture

A 35-year-old female was diagnosed with bilateral subluxated lenses and underwent successful vitrectomy, phacoemulsification, and IOL intrascleral fixation for the left eye. After 3 months, the patient complained of a dull pain in the left eye and was diagnosed with iris capture of the IOL. Tropicamide and pilocarpine drops were given at 15-min intervals, and the iris capture was soon alleviated, but it reappeared within a few days. The IOL was subsequently repositioned to the pars plana to achieve a greater separation of the IOL and iris. Iris capture reappeared 1 month after the surgery, as shown in Fig. 3a. After 21 months, rectangular loop suture surgery was performed for the left eye. The corrected visual acuity was 0.4 (− 2.25/− 2.00 × 5) preoperatively and improved to 0.5 one month after the surgery. Large astigmatism (− 1.00/− 5.50 × 165) appeared after the surgery, with the astigmatism axis almost perpendicular to the suture direction. The astigmatism decreased to 2.75 D at the one-month visit and to 2.25 D at the 9-month visit.

### Case 1-right eye

**Fig. 4:**
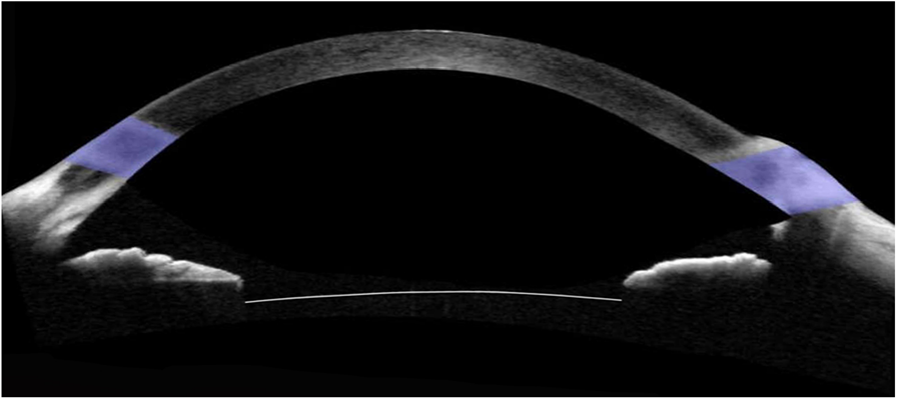
Postoperative anterior segment optical coherence tomography image. The curved white line indicates the anterior interface of the intraocular lens

The patient was similarly diagnosed with iris capture in the right eye 17 months after vitrectomy, phacoemulsification, and IOL intrascleral fixation. Rectangular loop suture surgery was performed on this eye 20 months later. Figure 4 shows the postoperative ultrasound biomicroscopy image of the right eye. The corrected visual acuity improved from 0.5 (− 3.00/− 1.50 × 180) to 0.6 (− 3.00/− 1.25 × 170) at the one-month visit.

### Case 2

A 72-year-old female who complained of distending pain in the right eye with accompanying nausea for half a month was diagnosed with iris capture of the IOL. The patient had undergone vitrectomy, IOL removal, and IOL intrascleral fixation one year earlier for IOL complete luxation. The preoperative corrected visual acuity was 0.16 (− 4.50/− 1.00 × 175) and the preoperative intraocular pressure (IOP) was 14.6 mmHg. We performed a rectangular loop suture on this patient. The suture was applied in the directions of 1:00 to 8:30 o’clock and 2:30 to 7:00 o’clock. The visual acuity was maintained at 0.16 (− 4.50/− 2.00 × 175) 3 months later. No recurrence was observed post surgery.

## Discussion

Iris capture is a common complication of IOL implantation for eyes with a capsule defect. It may be related to pupil dilation, IOL deviation or tilt, a floppy iris, or reverse pupillary block [8–10]. Mydriasis and subsequent miosis can release the iris capture, but recurrence is not uncommon, as seen in the right eye of case 1. Choi et al. [12] reported laser iridotomy as one treatment for iris capture, however, with only a 60% success rate. Moreover, surgical techniques are available for failed cases, which include the previously described strategies of IOL repositioning and IOL exchange. In Choi et al.’s study, IOL repositioning was used in 10 eyes, but was successful in only 4 eyes. In one of the failed eyes, the IOL was subluxated and subsequently treated by refixation, but iris capture was still present. A similar case was presented in the present report (left eye of case 1). The IOL was moved from 2 mm behind the limbus to 3.5 mm by an intrascleral fixation technique. However, iris capture reoccurred after this surgery. This failure may have been related to the relaxed iris and increased pupil size under dark conditions. IOL exchange is not commonly used because of the resulting large injuries. Yoo et al. reported a new IOL transscleral suture fixation technique, which they named the H technique [15]. The suture forms a barrier between the lens and the iris to avoid postoperative iris capture, the incidence of which was 2.5%. However, a complication of their technique must be considered, whereby the direction of the H suture was fixed and unchangeable after IOL fixation, such that iris capture may occur at the point where no suture passes.

The new rectangular loop suture technique prevents iris recapture by forming a barrier between the IOL optic and the iris, which is similar to H technique. However, with a well-designed suture direction, our technique is more individualized and precise. The applied direction was calculated according to the appearance of the iris capture before the surgery, as shown in Fig. 1. In case 1, we found that the iris capture tended to occur repeatedly in the same region of the iris, thus the suture was designed to pass behind this part of iris to prevent recurrence. However, more cases should be observed to verify this hypothesis. This technique can also correct the IOL rotation around the coronal axis by the contact between sutures and IOL optic. However, it is incapable to correct the other types of IOL dislocation.

Rho et al. [16] evaluated the influence of across-pupil sutures on the IOL optical quality and found no difference with or without sutures. In the present cases, the distance between two sutures was 3–4 mm, avoiding the central region of the pupil, thus no abnormal vision problems occurred post surgery.

During the operation, adequate attention should be focused on the IOP. When the pressure is insufficient during suturing, a large astigmatism will occur post surgery because of intraocular pressure recovery and the tightness of the suture. Astigmatism will decrease within several months, as shown in case 1.

## Conclusion

Our minimally invasive technique of rectangular loop suture can efficiently solve the complication of iris capture and is easy to perform.

## Data Availability

The datasets analysed during this study are available from the corresponding author on reasonable request.

## Abbreviations

IOL: Intraocular lens
IOP: Intraocular pressure
